# Quantification of basal stem cell elongation and stress fiber accumulation in the pseudostratified airway epithelium during the unjamming transition

**DOI:** 10.1242/bio.059727

**Published:** 2023-04-20

**Authors:** Thien-Khoi N. Phung, Jennifer A. Mitchel, Michael J. O'Sullivan, Jin-Ah Park

**Affiliations:** ^1^ Department of Environmental Health, Harvard T.H. Chan School of Public Health, Boston, MA 02115, USA; ^2^ Department of Biology, Wesleyan University, Middletown, CT 06459, USA

**Keywords:** Airway epithelium, Migration, Basal stem cell

## Abstract

Under homeostatic conditions, epithelial cells remain non-migratory. However, during embryonic development and pathological conditions, they become migratory. The mechanism underlying the transition of the epithelial layer between non-migratory and migratory phases is a fundamental question in biology. Using well-differentiated primary human bronchial epithelial cells that form a pseudostratified epithelium, we have previously identified that a confluent epithelial layer can transition from a non-migratory to migratory phase through an unjamming transition (UJT). We previously defined collective cellular migration and apical cell elongation as hallmarks of UJT. However, other cell-type-specific changes have not been previously studied in the pseudostratified airway epithelium, which consists of multiple cell types. Here, we focused on the quantifying morphological changes in basal stem cells during the UJT. Our data demonstrate that during the UJT, airway basal stem cells elongated and enlarged, and their stress fibers elongated and aligned. These morphological changes observed in basal stem cells correlated to the previously defined hallmarks of the UJT. Moreover, basal cell and stress fiber elongation were observed prior to apical cell elongation. Together, these morphological changes indicate that basal stem cells in pseudostratified airway epithelium are actively remodeling, presumably through accumulation of stress fibers during the UJT.

## Teaser

**Summary:** Quantitative analysis of the pseudostratified airway epithelium reveals basal stem cells and their stress fibers remodel during an unjamming transition.

## INTRODUCTION

Epithelial cells lining the large airway are complex in their differentiated cellular composition and form a pseudostratified columnar epithelium (Montoro et al., 2020). The pseudostratified human airway epithelium is recapitulated *in vitro* by culturing airway basal stem cells isolated from the human airway and then differentiated in air-liquid interface (ALI) culture (Gray et al., 1996; O'Sullivan et al., 2021). During ALI culture, epithelial layer maturation is marked by a decrease in cellular migration speed across the layer (Park et al., 2015). As the basal stem cells progressively differentiate through ALI days, the confluent layer transitions from a migratory unjammed phase towards a non-migratory jammed phase; this process is termed the jamming transition. When this jammed layer is exposed to pathologic stimuli, such as mechanical compression or ionizing radiation, epithelial cells become migratory again (Mitchel et al., 2020; O'Sullivan et al., 2020a; Park et al., 2015); this process is termed the unjamming transition (UJT). Both stimulations that we have previously shown to induce the UJT are related to pathologic conditions of the lung. Mechanical compression mimicking bronchospasm *in vitro* has been shown to induce asthma-associated mediators of airway remodeling (Kılıç et al., 2020; Lan et al., 2018; O'Sullivan et al., 2020b; Park et al., 2015; Veerati et al., 2020). Ionizing radiation causing DNA damage is known to induce pulmonary fibrosis (Xavier et al., 2004).

Unlike during the partial epithelial-to-mesenchymal transition (pEMT), a well-studied mechanism for achieving collective cellular migration, during the UJT the epithelium maintains its pseudostratified structure and barrier integrity (Mitchel et al., 2020). Epithelial migration is critical for physiological and pathologic conditions, including embryonic development, cancer metastasis, and tissue repair. In particular, the UJT has been implicated in airway branching morphogenesis as well as in chronic lung diseases (Park et al., 2015; Spurlin et al., 2019; Stancil et al., 2021, 2022). However, the underlying mechanism for collective migration during UJT in pseudostratified epithelia remains unknown.

For collective cellular migration in a simple columnar epithelium, cells generate traction forces on their underlying substrate through remodeling of their actin stress fibers. These forces are transmitted between neighboring cells and across the epithelial layer predominantly in the plane of migration (Kim et al., 2013; Tambe et al., 2011; Trepat et al., 2009). These intercellular forces drive collective cellular migration and are measurable using traction force microscopy and monolayer stress microscopy (Butler et al., 2002; DeCamp et al., 2020; Saraswathibhatla and Notbohm, 2020; Saraswathibhatla et al., 2020; Tambe et al., 2013; Trepat et al., 2009). For example, Saraswathibhatla and colleagues demonstrated that traction forces measured by traction force microscopy are increased in MDCK cells during the UJT (Saraswathibhatla and Notbohm, 2020; Saraswathibhatla et al., 2021). Moreover, their data indicate that increased traction force is associated with alignment of stress fibers. However, the traction and intercellular forces measured in a simple columnar epithelium, MDCK cells, cannot be intuitively extrapolated for a pseudostratified epithelium. The pseudostratified epithelium, such as that found in the airway, is composed of basal stem cells positioned underneath well-differentiated luminal cells. These well-differentiated luminal cells project mainly to the apical surface but are believed to have a relatively small footprint connecting to the underlying basement membrane (Evans et al., 2001). Therefore, during collective migration, the interactions between the apical and basal cells in pseudostratified epithelia remain challenging to decipher. In addition, studies from simple columnar epithelium indicate the significance of traction forces at the cell-substrate interface (Kim et al., 2013; Tambe et al., 2011; Trepat et al., 2009). However, in a pseudostratified epithelium it is unknown whether apical or basal cells generate forces needed to initiate collective migration. This knowledge gap limits our understanding of the physical mechanisms regulating the UJT.

Using pseudostratified human bronchial epithelium, we previously reported that collective cellular migration through the UJT is constrained by geometric elongation of the cells (Atia et al., 2018; Mitchel et al., 2020; O'Sullivan et al., 2020a; Park et al., 2015). In our previous studies, we exclusively measured cell shapes at the apical surface. Due to the complexity of the physical structure of the pseudostratified epithelium, the source of intracellular force generation leading to apical cell elongation remains unidentified. While traction forces and intercellular stresses can be measured by traction force microscopy, these methods are not compatible with the pseudostratified epithelium grown in ALI culture. In our previous work using a computational model simulating two-dimensional epithelial cell boundaries, we predicted that an increase in propulsive forces induces UJT. Our computational model predicted both cell elongation and increased migration speed, similar to those experimentally observed from phase-contrast microscopy of the pseudostratified epithelium (Mitchel et al., 2020). In pseudostratified epithelium, propulsive forces can be propagated by traction forces generated at the interface of the substrate and basal stem cells. Thus, we hypothesize that basal cells are a source of the traction forces during the UJT. As the first step to test our hypothesis and to extend our previous observations, we quantified the aspect ratio and area of basal stem cells and the length and alignment of actin stress fibers after inducing the UJT by two independent stimuli: mechanical compression and ionizing radiation. Our data reveal that basal stem cells elongated and enlarged, and their stress fibers elongated and aligned during the UJT. While our data are observational, these data provide a compelling rationale for further studies investigating the connection between cell shape changes and intercellular force generation during the UJT in pseudostratified epithelium.

## RESULTS

### During the unjamming transition, basal cells enlarge and elongate

**Fig. 1.**
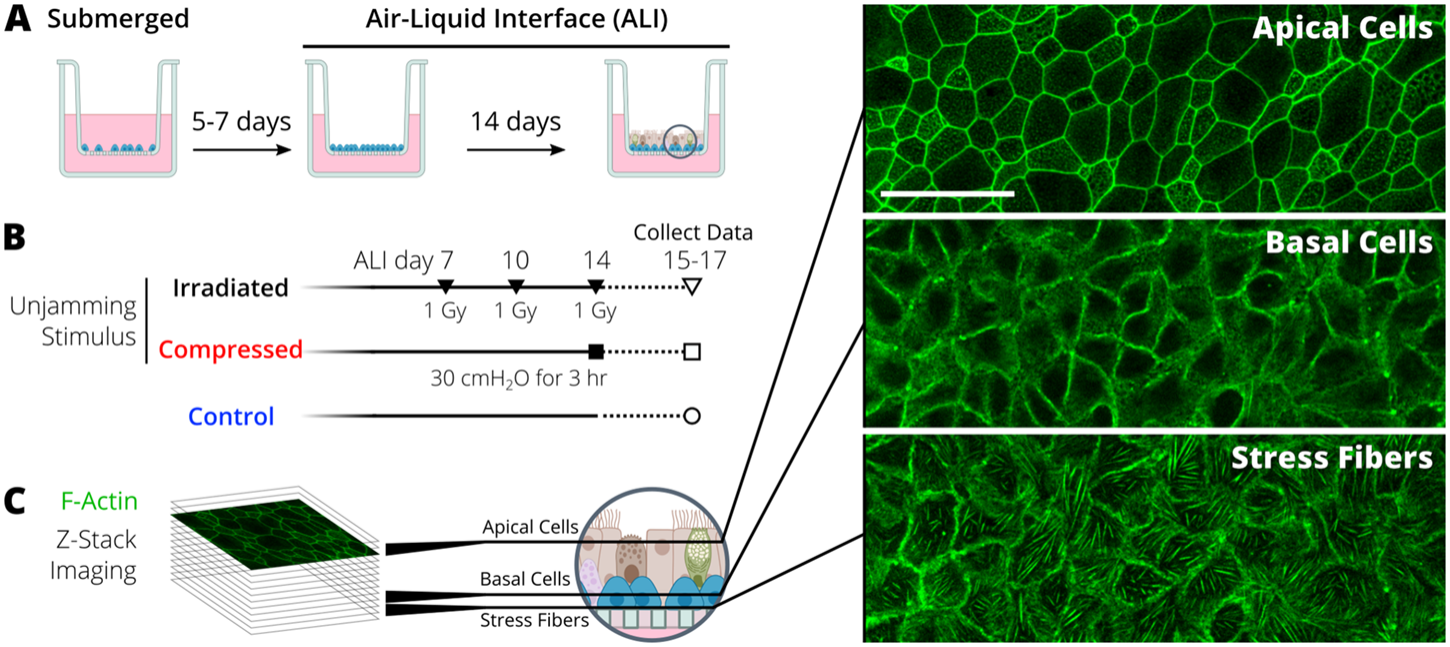
**Outline of the experiment for visualizing cells and stress fibers in the pseudostratified epithelium during the unjamming transition (UJT).** (A) Primary human bronchial epithelial cells were grown in submerged culture and then differentiated in air-liquid interface (ALI) conditions. (B) The jammed layer was exposed to either compression or irradiation to induce UJT. Imaging data were collected from the cells at 24, 48, and 72 h after the final stimulus (on ALI days 15, 16, and 17). (C) F-actin was stained and imaged in a z-stack. Maximum intensity projections were created to visualize different regions of interest through the apicobasally polarized epithelium. Scale bar: 50 µm.

To determine cell shape changes in the pseudostratified epithelium during the UJT, we used an *in vitro* system of human bronchial epithelial (HBE) cells differentiated in air-liquid interface (ALI) culture as previously reported (Fig. 1A) (Mitchel et al., 2016, 2020; Park and Tschumperlin, 2009; Park et al., 2015; Tschumperlin et al., 2003, 2004). To induce UJT, we exposed HBE cells to mechanical compression (Fig. 1B). To visualize cell morphology, we acquired z-stacks of immunofluorescence (IF) images of cells stained for F-actin (Fig. 1C, detailed in Materials and Methods). F-actin staining visualized both apical and basal cell boundaries as well as stress fibers. While cell boundaries marked by cortical actin were prominent through all focal planes, stress fibers were only detectable at the focal plane closest to the substrate, below the nuclei of basal cells. Within the apicobasally polarized epithelium, the regions distinct for apical cells, basal cells, and basal stress fibers were visualized using maximum intensity projections. To trace cell boundaries, we used marker-controlled watershed segmentation implemented in MATLAB (R2021a, Natick, MA, USA).

**Fig. 2.**
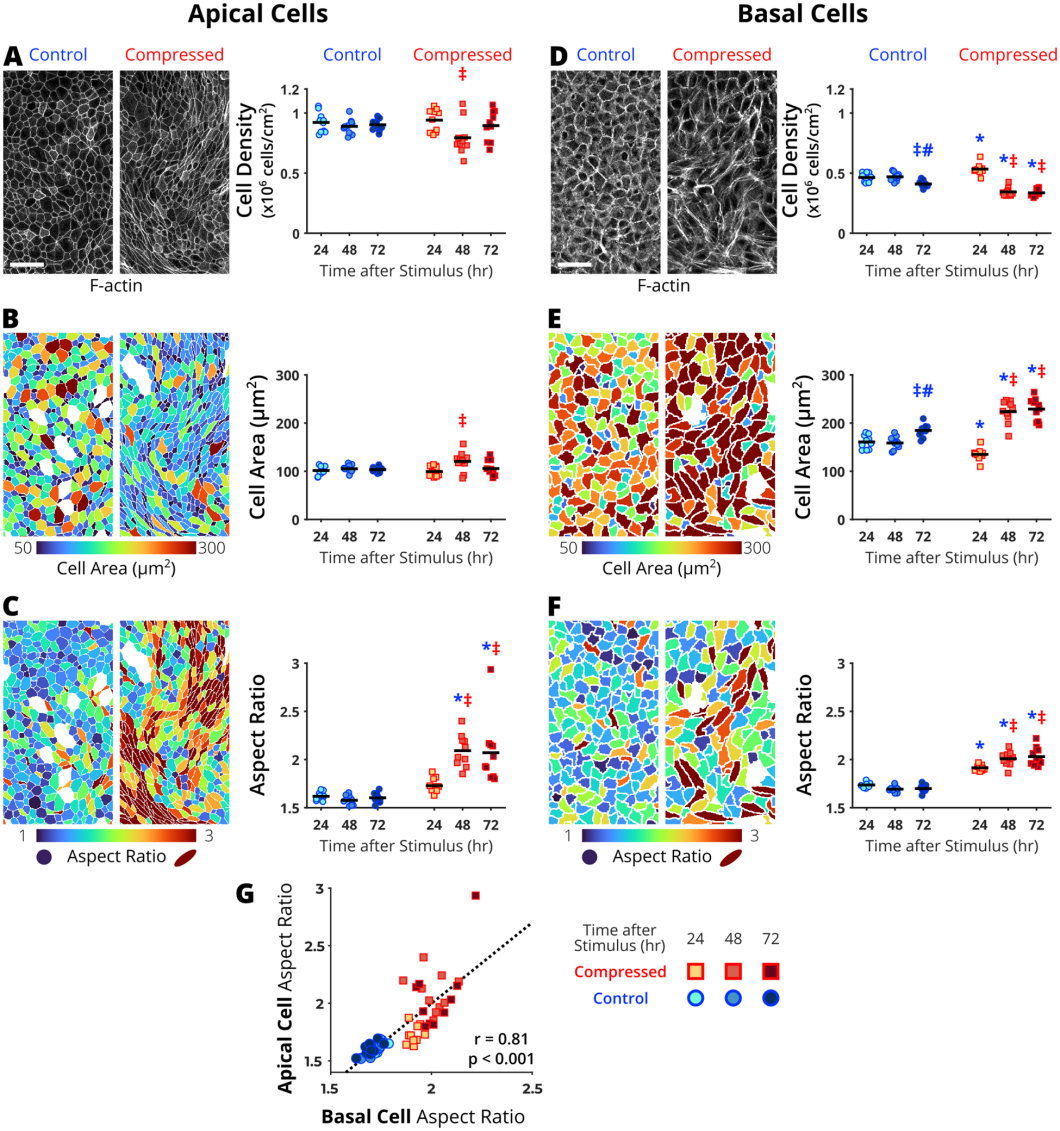
**In the pseudostratified airway epithelium, apical and basal cells undergo morphological changes during mechanical compression-induced unjamming transition (UJT).** The representative maximum intensity projections acquired from cells stained for F-actin and cell metric heatmaps are from 72 h after control or mechanical compression. During the UJT, apical cells remained constant in cell density (A) and in cell area (B), but became elongated (C). Basal cells decreased in cell density (D), increased in cell area (E), and became elongated (F). (G) At all measured timepoints, apical and basal cell elongation were significantly correlated. Each data point represents the average value over a field of view (*n*=10 fields of view per time point and treatment). Significant differences are indicated for *P*<0.05 from one-way ANOVA with Tukey-Kramer post-hoc test (*different from time-matched control, ^‡^different from 24 h within treatment group, ^#^different from 48 h within treatment group). Correlations were calculated using Pearson correlation coefficient (r). Scale bars: 50 µm.

To confirm the compressed epithelium became unjammed, we measured apical cell shape change, a previously identified hallmark of UJT (Atia et al., 2018; Mitchel et al., 2020; O'Sullivan et al., 2020a; Park et al., 2015). We also previously reported that UJT in HBE cells is independent of cell density. Thus, using the segmented apical cell boundaries, we first quantified apical cell density (Fig. 2A). In the control jammed layer, cell density remained constant over 72 h. At 48 h after compression, apical cell density decreased, but it was not statistically significant between jammed (control) and unjammed (compressed) epithelium. Next, we measured apical cell area (Fig. 2B). In the control jammed layer, cell area remained constant through 72 h. At 48 h after compression, cell area was enlarged, but it was not statistically significant between jammed (control) and unjammed (compressed) epithelium. Lastly, we measured apical cell aspect ratio (AR) to validate cell shape change as a hallmark of UJT (Fig. 2C). In the control jammed layer, AR remained constant over 72 h. After compression, AR significantly increased by 48 h (control: 1.58±0.05 versus compressed: 2.09±0.17, *P*<0.05). This compression-induced apical cell elongation was sustained through 72 h. Consistent with our previous reports, our new analysis of apical cell shape changes demonstrates that compression-induced UJT was independent of changes in apical cell density and area over 72 h and marked by significant apical cell elongation.

Using the same IF z-stack images where we detected apical cell elongation, we characterized changes in the basal stem cells (Fig. 1C). In pseudostratified epithelia, cell boundaries visualized at the apical surface feature differentiated cells, while cell boundaries visualized at the basal-substrate interface feature mostly undifferentiated basal stem cells (Fig. 1C) (Evans et al., 2001; Rock et al., 2010). Despite the critical role as epithelial stem cells during development and repair, their physical characteristics during cellular migration have not been studied. As the first step of gaining insights toward physical characteristics of basal stem cells, we characterized morphological changes in these basal cells using the same metrics applied to the apical cells. Basal cell boundaries were visualized approximately 13±3 µm below the apical cell surface (towards the substrate, Fig. 1C). From the segmented basal cell boundaries, we first quantified cell density (Fig. 2D). In the control jammed layer, basal cell density decreased at 72 h compared to the earlier time points (either at 24 or 48 h). In the compressed unjammed layer, basal cell density first increased at 24 h then significantly decreased at 48 and 72 h compared to time-matched jammed control. Next, we measured cell area (Fig. 2E). In the control jammed layer, basal cell area enlarged at 72 h. In the compressed unjammed layer, basal cell area decreased at 24 h (control: 161±14 versus compressed: 135±13 µm^2^, *P*<0.05). Basal cells then significantly enlarged by 48 h (224±24 µm^2^) and plateaued through 72 h (229±23 µm^2^). Together, these quantitative data indicate that basal cells are decreasing their cell density, while enlarging their cell area at the substrate interface during the UJT. The changes in basal cell density and area were unexpected and contrary to the apical cells, which recovered cell density and area similar to time-matched controls by 72 h after compression.

Similar to the apical cells, we characterized basal cell shape change by calculating AR (Fig. 2F). In the control jammed layer, AR of basal cells remained constant over 72 h. In the compressed unjammed layer, AR significantly increased by 24 h (control: 1.73±0.03 versus compressed: 1.92±0.03, *P*<0.05). AR further significantly increased by 48 h (2.01±0.07) and reached a plateau by 72 h (2.03±0.09). Additionally, basal cell elongation was highly correlated with apical cell elongation (r=0.81, *P*<0.001, Fig. 2G). Together, our quantitative analysis of basal cell elongation and enlargement indicates that apical cell elongation during the UJT was accompanied by basal stem cell remodeling.

### Stress fiber accumulation during the unjamming transition

**Fig. 3.**
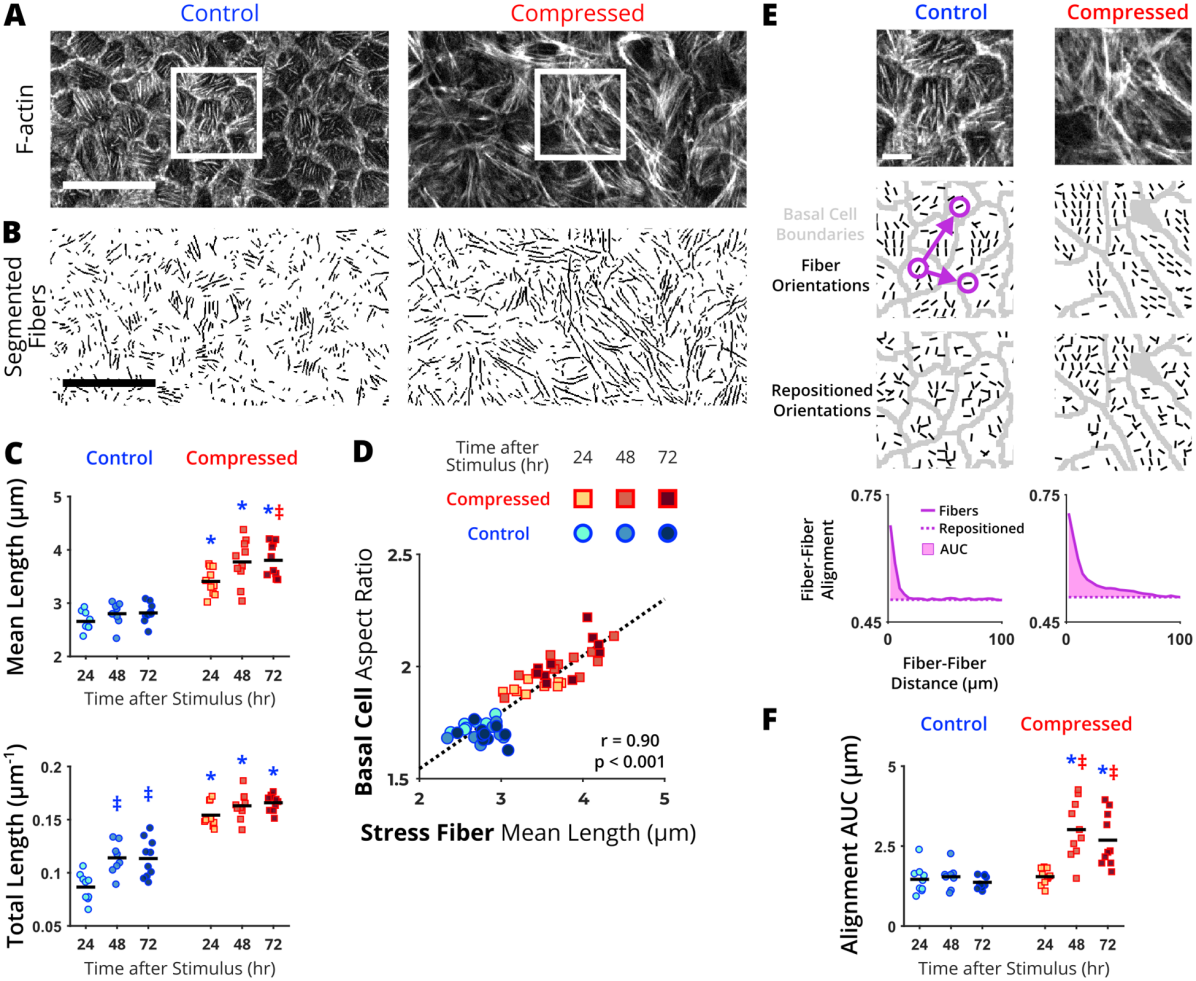
**Quantification of stress fiber elongation and alignment during the unjamming transition (UJT).** (A) In a focal plane below the basal cell boundaries, we visualized stress fibers marked by F-actin staining. These representative images from control jammed and compressed unjammed epithelium were taken at 72 h after UJT stimulus. (B) Stress fiber lengths were segmented. (C) Mean fiber lengths and total lengths all increased during the UJT. (D) Basal cell elongation and stress fiber elongation were significantly correlated. (E) To quantify stress fiber alignment, orientation fields were determined within segmented basal cell boundaries. The regions shown in (E) are indicated the inset in (A). Fiber alignment was analyzed by plotting the average alignment between pairs of fibers against the distance between them (e.g. purple circles and arrows) for both the experimental and repositioned orientations and measuring the area under the curve (AUC, detailed in Materials and Methods). (F) Alignment AUC increased during the UJT. Each data point represents the average value over a field of view (*n*=10 fields of view per time point and treatment). Significant differences are indicated for *P*<0.05 from one-way ANOVA with Tukey–Kramer post-hoc test (*different from time-matched control, ^‡^different from 24 h within treatment group, ^#^different from 48 h within treatment group). Correlations were calculated using Pearson correlation coefficient (r). Scale bars: 50 µm in A and B; 10 µm in E.

Our data indicate that basal stem cells in pseudostratified epithelia change both their shape and size during the UJT. These changes often reflect perturbations in cellular mechanics. To build the link between shape changes and cellular mechanics during the UJT, we examined stress fiber accumulation in the basal cells. Stress fibers generate traction forces, which modulate cellular mechanics (Burridge and Guilluy, 2016; Sala and Oakes, 2021; Walcott and Sun, 2010; Zhang et al., 2017). In a simple columnar epithelium modeled by MDCK cells, unjamming marked by cell shape and migration is controlled by stress fibers that generate traction forces at the interface between cell and substrate (Saraswathibhatla and Notbohm, 2020). Furthermore, stress fiber alignment has been shown to integrate cytoskeletal forces to drive directed cell migration (Fischer et al., 2021). However, both qualitative and quantitative analyses on stress fiber accumulation have not been reported in pseudostratified epithelia during the UJT. To gain insight into the physical forces exerted by stress fibers during the UJT in the pseudostratified epithelium, we visualized actin stress fibers 2.2±0.4 µm below the focal plane where basal cell boundaries were visualized (Fig. 1C). We assessed both qualitative and quantitative differences in basal stress fibers between control jammed and compressed unjammed layers (Fig. 3A). The qualitative difference of stress fiber can be described by spatial distributions within the layer. In the control jammed layer, stress fibers were confined within each of the cell boundaries prominently marked by cortical actin. However, in the compressed unjammed layer, stress fibers spanned multiple cell lengths and the cell boundaries marked by cortical actin were less prominent.

For the quantitative assessment of stress fibers, we segmented fiber lengths using a combination of two published algorithms, FSegment (Rogge et al., 2017) and Stress Fiber Extractor (SFEX) (Zhang et al., 2017). In the control jammed layer, we masked the visible cell boundaries to exclude potential segmentation of cortical actin and to segment solely the stress fibers (Fig. 3B). We calculated the mean length of individual fibers and total length per each field of view. In the control jammed layer, mean stress fiber lengths remained constant over time (Fig. 3C). However, the total length of stress fibers increased over time (24 h: 0.09±01 and 72 h: 0.11±0.01 µm^−1^, *P*<0.05). In the compressed unjammed layer, mean stress fiber lengths increased at 24 h (control: 2.7±0.2 versus compressed: 3.41±0.24 µm, *P*<0.05), continued to increase at 48 h, and plateaued by 72 h. Similarly, total stress fiber lengths increased at 24 h (control: 0.09±0.01 µm^−1^ versus compressed: 0.15±0.01 µm^−1^, *P*<0.05) and continued to increase over 72 h. Our data indicate that stress fibers were significantly elongated and accumulated during the UJT. Moreover, the time course of stress fiber elongation was significantly correlated with basal cell elongation (r=0.90, *P*<0.001) (Fig. 3D).

To characterize organization of stress fibers, we used the software MatFiber that estimates stress fiber orientations (Fig. 3E, detailed in Materials and Methods) (Fomovsky and Holmes, 2010). To quantify regional heterogeneity of alignment, we used the orientation vector fields (Richardson and Holmes, 2016). In the control jammed layer, the stress fibers maintain their heterogeneous alignment, measured using alignment area under the curve (AUC) (Fig. 3F). In the compressed unjammed layer, alignment AUC significantly increased by 48 h (control: 1.5±0.4 versus compressed: 3.0±0.9 µm^−1^, *P*<0.05). The increased alignment was sustained through 72 h of the UJT. Together, our data demonstrate that during compression-induced UJT, stress fibers elongated and aligned in basal cells.

### Basal cell elongation and stress fiber accumulation are novel hallmarks of the unjamming transition

**Fig. 4.**
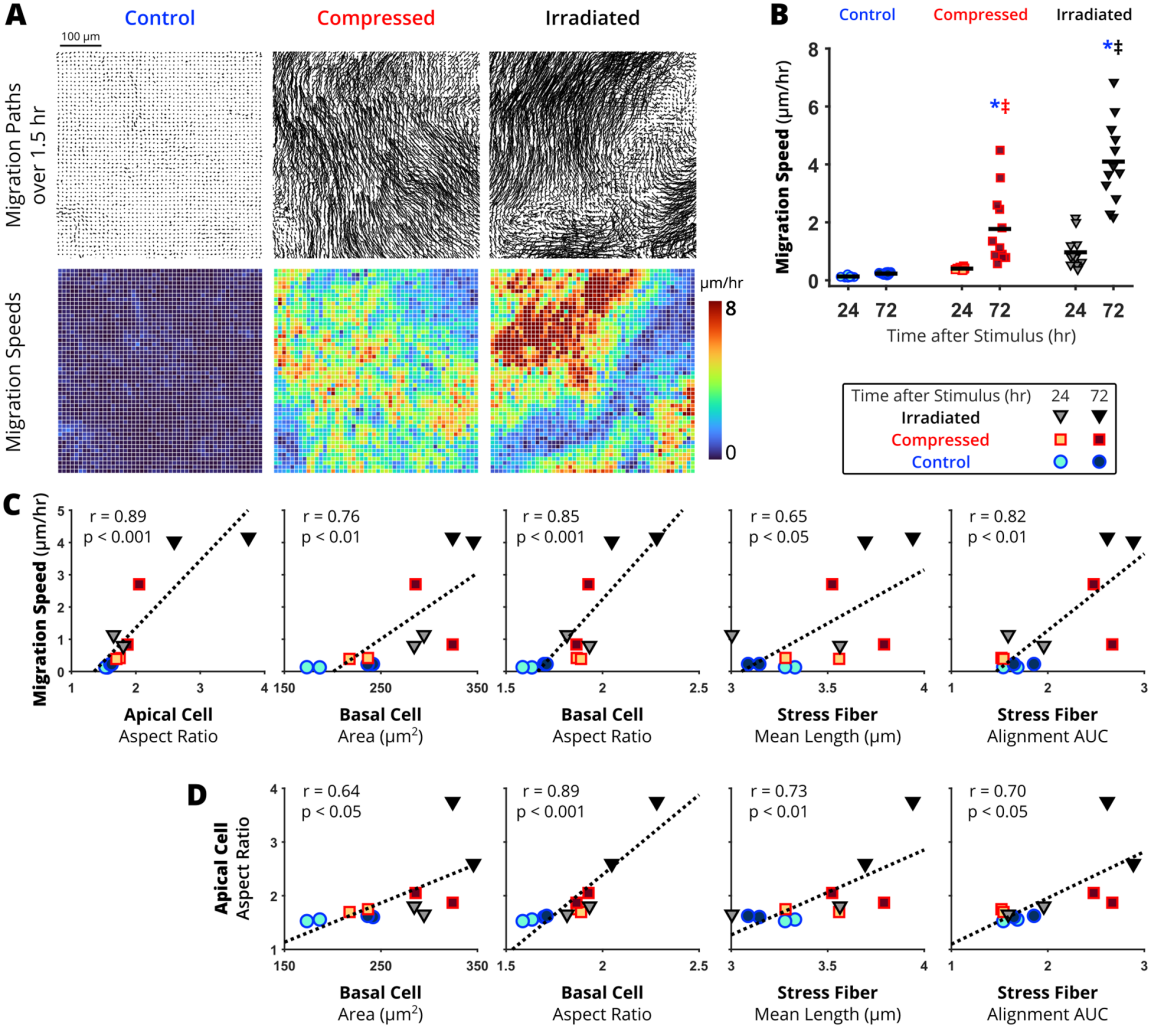
**Metrics of basal cell remodeling correlate with migration speed and apical cell elongation during the unjamming transition.** Unjamming was induced by either compression or irradiation. At 24 or 72 h after exposure to either stimulus, time-lapse phase microscopy images were taken to calculate migration speed. (A) In representative fields of view which were taken at 72 h, migration paths starting from a 10 µm grid were traced to calculate speeds over 1.5 h. (B) At 24 and 72 h, the average migration speed was calculated over each field of view for the jammed (control) and unjammed (compressed and irradiated) layers. Significant differences are indicated for *P*<0.05 from one-way ANOVA with Tukey–Kramer post-hoc test (*different from time-matched control, ^‡^different from 24 h within treatment group, ^#^different from 48 h within treatment group). (C) Migration speeds were correlated with apical cell elongation, basal cell enlargement and elongation, as well as stress fiber elongation and alignment. (D) Apical cell elongation was correlated with basal cell enlargement and elongation as well as stress fiber elongation and alignment. Each data point in (C,D) represents the average across one experimental well. Correlations were calculated using Pearson correlation coefficient (r) with *n*=2 wells per treatment and timepoint.

With our data revealing significant correlations between apical cell elongation, basal cell elongation, and stress fiber accumulation during compression-induced UJT, we further validated if these changes are common characteristics of the UJT regardless of the stimuli. To induce UJT, we exposed the cells to either mechanical compression or ionizing radiation (Fig. 1B). After inducing UJT by either stimulus, we measured both dynamic (cell migration speed) and structural characteristics of the UJT. As in previous work, both mechanical compression and ionizing radiation induced collective cellular migration after 72 h as visualized by spatially coordinated migration paths and speeds (Fig. 4A). In the control jammed layer, the migration paths over a 1.5-h period remained centered on the initial tracking grid. After induction of UJT by either compression or irradiation, the migration paths exhibited dramatic swirling patterns with distinct clusters of high and low speeds indicative of cellular unjamming (Angelini et al., 2011; Mitchel et al., 2020; Palamidessi et al., 2019; Park et al., 2015; Vishwakarma et al., 2020). In the control jammed layer, the average speed of migration remained low (24 h: 0.13±0.03 and 72 h: 0.23±0.04 µm/h, Fig. 4B). After compression or irradiation, the average speed of cell migration significantly increased by 72 h (compressed: 1.77±1.25 µm/h; irradiated: 4.10±1.42 µm/h).

We calculated the correlation between the newly characterized basal cell and stress fiber morphology metrics and two previously published hallmarks of the UJT (migration speed and apical cell elongation) (Mitchel et al., 2020; Park et al., 2015). During the UJT, migration speed was significantly correlated with apical cell elongation (r=0.89, *P*<0.001) as well as the following newly characterized structural metrics: basal cell enlargement (r=0.76, *P*<0.01), basal cell elongation (r=0.85, *P*<0.001), stress fiber elongation (r=0.65, *P*<0.05), and stress fiber alignment (r=0.82, *P*<0.01) (Fig. 4C). Additionally, apical cell elongation was correlated with the newly characterized structural metrics, including basal cell enlargement (r=0.64, *P*<0.05), basal cell elongation (r=0.89, *P*<0.001), stress fiber elongation (r=0.73, *P*<0.01), and stress fiber alignment (r=0.70, *P*<0.05) (Fig. 4D). The significant correlation of basal cell and stress fiber metrics with migration speed and apical cell elongation, in response to two independent UJT stimuli, support basal cell and stress fiber remodeling as novel structural hallmarks of the UJT in pseudostratified epithelium.

## DISCUSSION

Collective cellular migration is a critical cellular process in development and disease (Crosby and Waters, 2010; Goodwin and Nelson, 2021; Varner and Nelson, 2014). One of the known mechanisms of collective migration of epithelial cells is through the UJT (Atia et al., 2021; Buttenschön and Edelstein-Keshet, 2020; Garcia et al., 2015; Kim et al., 2020; La Porta and Zapperi, 2020; Oswald et al., 2017; Palamidessi et al., 2019; Park et al., 2016). The hallmarks of the UJT in pseudostratified airway epithelium have been previously characterized by collective cellular migration and apical cell elongation (Atia et al., 2018; Mitchel et al., 2020; Park et al., 2015). While these characteristics of the UJT suggest the presence of intercellular force changes, the cell-specific contributions and mechanisms of physical forces driving collective cellular migration in the UJT are unknown.

Despite the heterogeneity of pseudostratified airway epithelium composed of both differentiated apical cells and undifferentiated basal stem cells, the cell-type specific characteristics during UJT have not been studied. In pseudostratified airway epithelia, multiple types of differentiated epithelial cells are in contact with the basal lamina (or substrate *in vitro*); however, basal stem cells occupy most of the basal lamina area (Evans et al., 2001). Because of this prominent contact, we hypothesized that basal cells generate tractions forces necessary for migration during the UJT. Because measuring traction forces is challenging in ALI cell culture systems, we interrogated the role of basal cells in the UJT using image analysis to characterize morphological changes in basal cells and their stress fibers. Our results reveal that basal stem cells actively remodel as part of the UJT in pseudostratified airway epithelium.

In our new quantitative analysis of basal stem cells in pseudostratified airway epithelium, our data unveil four novel hallmarks of the UJT: basal cell enlargement and elongation as well as stress fiber elongation and alignment. By 24 h of UJT, basal cells elongated (Fig. 2E) and their stress fibers elongated (Fig. 3C). By 48 h, basal cells enlarged (Fig. 2F) and their stress fiber aligned (Fig. 3F). Together, our data demonstrate that basal cells and their stress fibers actively remodel during the UJT. Importantly, basal cell remodeling preceded apical cell elongation and increased collective cellular migration speed (at 48 h, Fig. 2B), previously established hallmarks of the UJT (Atia et al., 2018; Mitchel et al., 2020; Park et al., 2015). In our previous studies, we observed apical cell elongation and collective cell migration are significantly increased by 24 h after compression. The delay in apical cell elongation in the current data set may be due to donor-to-donor variability as widely observed in primary HBE cells (Park et al., 2015).

During the UJT, apical cells maintained constant cell area and density (Fig. 2A and C); however, basal cells enlarged and decreased their density (Fig. 2D and F). These data may reflect previously reported observations of basal cell remodeling in pseudostratified airway epithelium in airway diseases or after injury. For example, airway basal cells flatten (or increase cell area) in response to damage and loss of differentiated cells above them to preserve barrier integrity (Erjefält et al., 1997; Puchelle et al., 2006). Accompanying the basal cell enlargement, our data indicate that basal cell density decreases during UJT; however, the mechanism for basal cell loss remains unclear. We speculate that basal cells may have been extruded during UJT. Or these basal stem cells may be differentiating to replace any loss of apical cells (through cell death or extrusion) (Baba et al., 2020; Gudipaty and Rosenblatt, 2017; Kretschmer et al., 2017; Tadokoro et al., 2016). In monolayer cell culture, increasing cell density has been shown to decrease the speed of collective cellular migration (Angelini et al., 2011; Garcia et al., 2015; Loza et al., 2016; Saraswathibhatla and Notbohm, 2020; Tambe et al., 2011; Tlili et al., 2018). However, in pseudostratified epithelium, changes in density were not previously explored or characterized during the UJT. Thus, our data demonstrating changes in basal cell density suggest differentiated cell composition may be important to study in the UJT.

We further validated the four novel hallmarks of the UJT in an independent dataset of images acquired from either compression- or irradiation-induced UJT (Fig. 4). Consistent with our previous report, both compression and irradiation induced UJT, but the extent of cell migration speed and apical cell elongation were greater in irradiation-induced UJT (Fig. 4B and C). These two previously established hallmarks of UJT were significantly correlated with the newly characterized basal cell remodeling metrics, including basal cell elongation and stress fiber accumulation (Fig. 4C and D). In our previous reports, we focused on cell shape changes at the apical surface. Here, our new observational data reveal that even before the onset of cellular migration or apical cell elongation, basal cells are actively remodeling during the UJT in pseudostratified airway epithelium. Therefore, how basal cells undergo remodeling and how they play a role during UJT should be further investigated to advance our mechanistic insight toward the UJT.

**Fig. 5.**
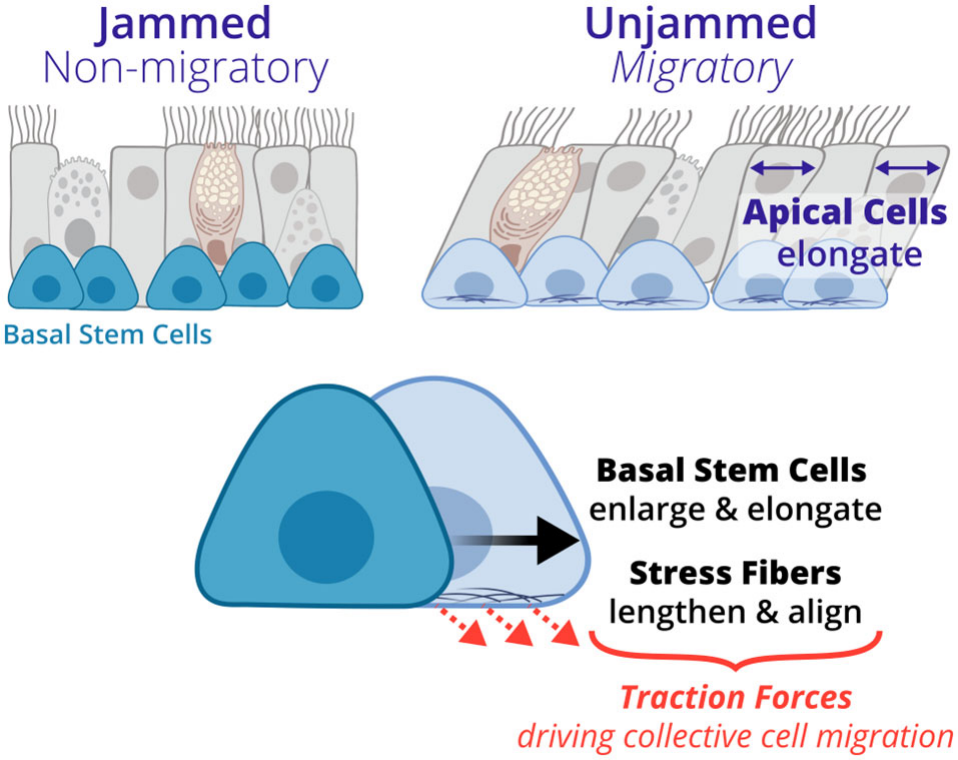
**A conceptual schematic of the unjamming transition (UJT) in pseudostratified airway epithelium.** UJT has been previously characterized by increased collective cellular migration and apical cell elongation (Park et al., 2015). In this study, we now identified that basal stem cells undergo unique structural changes, including cell elongation and enlargement as well as stress fiber elongation and alignment during the UJT. Our new quantitively analysis suggests that basal stem cells may generate traction forces exerted by stress fiber during collective cellular migration.

Quantifying how physical forces contribute to the migration process is critical to mechanistic understanding of the UJT. However, the physical forces driving UJT in pseudostratified epithelium are not well understood due to limitations in methods to directly measure traction forces in ALI culture. Prior computational modeling studies have suggested that increased cell–cell adhesion at the cell periphery can induce the UJT (Bi et al., 2014; Park et al., 2015, 2016). In the case of pseudostratified epithelium, UJT has been attributed to changes in cortical tension or adhesions at the cell boundaries; however, the observations supporting this mechanism have been measured exclusively at the apical surface (Mitchel et al., 2020; O'Sullivan et al., 2020a; Park et al., 2015). Computational modeling has also suggested that increasing cellular force generation (propulsive forces) in the model also induces the UJT in the absence of changes to cortical tension or adhesion (Mitchel et al., 2020). Experimentally, traction forces have been directly measured in a monolayer of cells and shown to be critical for cell-shape change and migration during UJT (Saraswathibhatla and Notbohm, 2020). Specifically, traction forces are generated by actin stress fibers and lead to cell elongation, suggesting the significance of forces at the cell-substrate interface. In the absence of traction force measurements, cell shape elongation and stress fiber accumulation and alignment have been demonstrated to be surrogate metrics for traction force generation (Saraswathibhatla and Notbohm, 2020; Vignaud et al., 2020). Our observational data indicate that in pseudostratified epithelia, basal cells and their stress fibers at the substrate interface undergo remodeling suggestive of traction force generation during the UJT (Fig. 5). These cellular forces induced by accumulated stress fibers may be the driving mechanism for the previously observed apical cell elongation and collective cellular migration. However, the physical mechanism connecting basal cell traction forces with apical cell elongation remains unclear.

Apical cells in a pseudostratified epithelium are tethered together via a combination of tight junctions and adherens junctions, and basal cells are tethered to the apical cells via desmosomes (Evans et al., 2001; Niessen, 2007; Roche et al., 1993; Rock et al., 2010). As the basal cells generate traction forces, desmosomes may be relaying these forces to the apical cells to induce the previously described hallmarks of the UJT (Mitchel et al., 2020; O'Sullivan et al., 2020a; Park et al., 2015). Desmosome assembly in a migrating cell monolayer has been shown to localize at the leading edge of migration and require actin cytoskeleton force generation (Koetsier et al., 2014; Roberts et al., 2011; Thomason et al., 2010). Additionally, the role of cell neighbors throughout the apicobasal axis of the epithelium may be critical to understanding intercellular force transmission and unjamming (Gómez et al., 2021; Grosser et al., 2021). The role of desmosomes in physical tethering of basal and apical cells as well as the three-dimensional organization of cells should be further studied in unjammed pseudostratified epithelium.

In this study, we performed quantitative analyses of basal stem cells and actin stress fibers. Our data reveal that basal cells actively remodel and may be the site of traction force generation during collective cellular migration during the UJT. We identified new hallmarks of the UJT: basal cell enlargement and elongation as well as stress fiber elongation and alignment. These hallmarks expand the library of metrics that can be used to characterize the jamming or unjamming of pseudostratified epithelium in both *in vitro* and *in vivo* models. Furthermore, this work supports future mechanistic studies towards manipulating basal stem cell remodeling to decipher their role in the UJT. While basal stem cells have been well-recognized as a rich source of biochemical signals as epithelial progenitor cells, our new findings recognize a potential critical, physical role for basal stem cells as an active participant in collective cellular migration.

## MATERIALS AND METHODS

### Culture of primary human bronchial epithelial cells

Primary human bronchial epithelial (HBE) cells were obtained from the Marsico Lung Institute/Cystic Fibrosis Research Center at the University of North Carolina at Chapel Hill (UNC) under protocol number 03-1396 approved by the UNC Biomedical Institutional Review Board. The two donors used in this study were from non-smokers with no history of chronic lung disease. As described previously (Mitchel et al., 2016, 2020; O'Sullivan et al., 2020a, 2021; Park and Tschumperlin, 2009; Park et al., 2010, 2012, 2015), HBE cells at passage 2 were cultured on transwell inserts (Corning, 12 mm, 0.4 µm pore, polyester) and maintained in air-liquid interface (ALI) for 14 days until well-differentiated (Fig. 1).

To induce the UJT, we exposed cells to either mechanical compression or ionizing radiation (Fig. 1) (Mitchel et al., 2020; O'Sullivan et al., 2020a; Park et al., 2015). For mechanical compression, cells were exposed to an apical-to-basal pressure differential of 30 cmH_2_O for 3 h on ALI day 14 (Mitchel et al., 2016, 2020; Park and Tschumperlin, 2009; Park et al., 2010, 2012, 2015; Tschumperlin et al., 2004). For irradiation, cells were exposed to a dose of 1 Gy of ionizing radiation using a RS 2000 Biological Research Irradiator (RadSource, Brentwood, TN) on ALI culture days 7, 10, and 14 (O'Sullivan et al., 2020a). Following the final unjamming stimulus on ALI day 14, cells were maintained in ALI culture for up to 72 h.

In the cells from donor one, we applied mechanical compression to induce the UJT and collected imaging data after 24, 48, or 72 h. In the cells from donor two, we induced unjamming using either mechanical compression or irradiation and collected imaging data 24 or 72 h after each of the unjamming stimuli. For all experiments, two transwells were imaged for each treatment and timepoint.

### Immunofluorescence staining and static imaging

At 24, 48, or 72 h after final exposure to the unjamming stimulus, cells were fixed with 4% paraformaldehyde in PBS with calcium and magnesium for 30 min at room temperature. Cells were permeabilized with 0.2% Triton X-100 for 15 min, blocked with 1% bovine serum albumin and 10% normal goat serum for 1 h, and stained for F-actin (Alexa fluor 488-Phalloidin, ThermoFisher Scientific, diluted 1:40, 30 min). Transwell membranes were cut from the plastic support and mounted on glass slides (Vectashield). Slides were imaged using a Zeiss Axio Observer Z1 with an apotome module controlled using Zen Blue 2.0 software. Five random fields of view (563 by 356 µm) were imaged from each transwell membrane in a z-stack from substrate to apical cell surface (15±3 µm in height).

To visualize various planes through the pseudostratified epithelial layer (Fig. 1), maximum intensity projections were generated in Fiji (Schindelin et al., 2012). The apical surface was generated from all z-stack slices that included visible apical boundaries. Approximately 13±3 µm below the apical surface, the basal cell boundaries were manually masked using a sliding region of interest across the field of view in sequential z-stack slices before a maximum intensity projection was generated. To visualize stress fibers, the identical regions of interest for the basal cells were masked in lower z-stack slices (2.2±0.4 µm) towards the transwell membrane before generating a maximum intensity projection.

### Cell shape analysis

Cell boundaries were visualized from maximum intensity projections of apical and basal surfaces, generated as described above, using marker-controlled watershed segmentation in MATLAB (R2021a). Apical cell images were first filtered using opening-closing by reconstruction. After initial watershed segmentation, quality control of the segmented cell borders was assessed by calculating boundary tortuosity, length, and mean pixel intensity. Over-segmented boundaries were removed based on thresholds of the three metrics. Similarly, basal cell images were first filtered using a Wiener filter followed by watershed segmentation. Apical and basal cell bodies were lastly filtered based on area to remove under- and over-segmented regions.

Cell morphology was characterized by measuring cell area and aspect ratio. Cell area was quantified in the plane parallel to the transwell membrane (Mitchel et al., 2020; O'Sullivan et al., 2020a; Park et al., 2015). Cell aspect ratio was defined as the ratio of the major- to minor-axis length for an ellipse fit to the segmented cell boundary. These metrics were mapped to the segmentation for visualization of morphology changes. For comparison between treatments and timepoints, cell morphology metrics were averaged over each field of view.

### Stress fiber analysis

Stress fibers lengths were segmented using a combination of two published algorithms: FSegment (Rogge et al., 2017) and Stress Fiber Extractor (SFEX) (Zhang et al., 2017). Briefly, the FSegment algorithm is designed to iteratively trace fiber fragments within an image (Rogge et al., 2017). The iterative design specifically overcomes the challenge of superimposed stress fibers. FSegment outputs fiber fragments which often separate longer fibers into multiple shorter regions. We used the fiber reconstruction algorithm developed in SFEX to connect the fragments. Briefly, SFEX evaluates the end points of all fiber fragments and connects them if they meet geometric constraints (based on distance and orientation of fiber fragments) (Zhang et al., 2017). Additionally, we added a constraint to check whether potential connecting segments between fiber fragments featured pixel intensities matching the fiber fragments. In the control groups, cortical actin at the cell boundaries confounded the stress fiber segmentation (Fig. 3A); therefore, we removed the basal boundary regions segmented from the basal cell plane image before segmenting fibers. In the unjammed groups, cortical actin was not prevalent; therefore, we did not mask the basal cell boundaries. To measure the mean length of stress fibers, we averaged the lengths across a field of view. To measure the total length of stress fibers, we summed the lengths and divided by the size of the field of view searched by the segmentation algorithm.

To analyze alignment of stress fibers, we quantified fiber orientations using the software MatFiber and measured spatial heterogeneity (Fomovsky and Holmes, 2010; Richardson and Holmes, 2016). To isolate the stress fibers from the cell boundaries, we removed the basal cell boundaries segmented from the basal plane image and dilated to a width of 1.5 µm (Fig. 3E). We evaluated the orientation of stress fibers in finite subregions (3 by 3 µm) of the image with MatFiber. MatFiber uses an intensity-gradient-detection algorithm to measure orientation of fibers. To exclude subregions without stress fibers, we removed subregions with mean pixel intensity below the 35th percentile based on the basal cell boundary-free image. To evaluate the spatial heterogeneity of stress fiber orientations, we calculated the Alignment Area Under the Curve (AUC) metric described in detail by (Richardson and Holmes, 2016). From the experimental orientation field, we plotted the average alignment (dot product) between pairs of fibers against the distance between them. We then randomly repositioned all the orientation vectors to remove local alignment and repeated the plotting. The area captured between the two curves (Alignment AUC) is a single measure that characterizes the degree to which local alignment of fibers exceeds global alignment across the image. Higher values of Alignment AUC indicate that stress fibers are more similarly aligned locally.

### Live imaging and cellular migration analysis

Cellular migration speeds were measured from time-lapse images taken at 24 or 72 h after unjamming stimulus treatment. For each independent experimental replicate (two transwells per treatment per timepoint), six fields of view (1124 by 713 µm) per well were imaged every 6 min over 1.5 h. The imaging chamber was supplied with 37°C, 5% CO_2_, humidified air on a Zeiss Axio Observer Z1 to collect phase contrast images. Flow fields were calculated using optical flow with the Farneback method in MATLAB (Farnebäck, 2003). A 10 by 10 µm grid was initially seeded in the first image, and the migration trajectories were calculated by forwards-integration of the flow field. The average migration speed was calculated from the displacement over the 1.5-h time lapse.

### Statistics

For each treatment and timepoint, two wells were imaged with five fields of view for z-stacks and six fields of view for time lapses. All statistics and visualizations were computed in MATLAB. To compare metrics across treatments and timepoints, we averaged the metric across each field of view and used a one-way ANOVA with Tukey–Kramer post-hoc test. Groups were considered statistically significant for *P*<0.05. For data visualization, we show a data point for each field of view with a solid line in each group to indicate the mean value. In the text, metrics are reported as mean±s.d.

For correlations between metrics, we calculated a Pearson correlation coefficient (*r*) and report the *P*-value. The correlations between cell morphology and stress fiber metrics (Figs 2G and 3D) used data points from matched z-stacks. The correlations with migration speeds (Fig. 4C) or apical cell aspect ratio (Fig. 4D) used data points averaged across each transwell. To get a single value for each transwell, the cell and stress fiber morphology metrics were averaged across five fields of view per transwell, and the migration speeds were averaged across six fields of view per transwell. For data visualization, we show the individual data points with a dashed line indicating the line of best fit.
